# Suicides on the Austrian railway network: hotspot analysis and effect of proximity to psychiatric institutions

**DOI:** 10.1098/rsos.160711

**Published:** 2017-03-08

**Authors:** Markus J. Strauss, Peter Klimek, Gernot Sonneck, Thomas Niederkrotenthaler

**Affiliations:** 1 Suicide Research Unit, Department of Social and Preventive Medicine, Center for Public Health, Medical University of Vienna, Kinderspitalgasse 15, 1090 Vienna, Austria; 2 Section for Science of Complex Systems, Medical University of Vienna, Spitalgasse 23, 1090 Vienna, Austria; 3 Crisis Intervention Centre, Lazarettgasse 14A, 1090 Vienna, Austria

**Keywords:** railway, suicide, prevention, cluster, hotspot, spatial point pattern, Austria

## Abstract

Railway suicide is a significant public health problem. In addition to the loss of lives, these suicides occur in public space, causing traumatization among train drivers and passengers, and significant public transport delays. Prevention efforts depend upon accurate knowledge of clustering phenomena across the railway network, and spatial risk factors. Factors such as proximity to psychiatric institutions have been discussed to impact on railway suicides, but analytic evaluations are scarce and limited. We identify 15 hotspots on the Austrian railway system while taking case location uncertainties into account. These hotspots represent 0.9% of the total track length (5916 km/3676 miles) that account for up to 17% of all railway suicides (*N*=1130). We model suicide locations on the network using a smoothed inhomogeneous Poisson process and validate it using randomization tests. We find that the density of psychiatric beds is a significant predictor of railway suicide. Further predictors are population density, multitrack structure and—less consistently—spatial socio-economic factors including total suicide rates. We evaluate the model for the identified hotspots and show that the actual influence of these variables differs across individual hotspots. This analysis provides important information for suicide prevention research and practice. We recommend structural separation of railway tracks from nearby psychiatric institutions to prevent railway suicide.

## 1. Background

Suicide is a considerable public health problem. According the World Health Organization [1], more than 1 million individuals die by suicide globally each year. In Western countries, suicide ranks consistently among the top three causes of death among young individuals, resulting in a tremendous social, psychological and economic burden to individuals, families and society. Railway suicide is a suicide method that involves the jumping or lying in front of a train in order to die by suicide. These suicides typically occur in public locations, thereby affecting and traumatizing a large number of individuals including train drivers, passengers and clean-up personnel. Moreover, railway suicides result in significant delays in public transport. For these reasons, these suicides have received a specific focus in suicide prevention initiatives [2–5]. Individuals dying by railway suicide have been found to be of younger age as compared to the average suicide decedents [6–8], more often involve individuals with severe mental illness [6,9,10], and suicide decedents typically reside close to the railway tracks [6,11].

Density-based clustering of railway suicides has been reported in the literature [7,12]. In particular, suicides seem to be more frequent close to psychiatric institutions [9,10,12], and also railway parameters including high train frequency have been found to be associated with railway suicides in some studies [13,14]. Some [11], but not all [7] studies suggest that railway suicides increase with increasing population density close to the railway. Media and social contagion effects may play a role in the formation of locations with high density of railway suicide events [15,16], so-called hotspots.

Studies on the clustering of suicides, in general, and on area-related factors that impact on railway suicides specifically have been hampered by a number of limitations. First, correlations between variables such as population density and railway suicides have often been assessed on an aggregate level over time, without looking at spatial variations of densities across the whole length of the railway track. Second, probable measurement errors regarding the specific locations of individual railway suicides have not been taken into account. Finally, models to estimate the relative influence of different factors have typically not controlled for both area-related population and railway factors, and have not been validated with adequate simulation techniques.

When it comes to the assessment of clustering on railway networks, an additional challenge in the field of suicide prevention research has been that any simulation needs to take into account that cases can only occur along the railroad network. Here, a careful consideration of distance measurement between cases and objects such as psychiatric institutions needs to be made: distance can be measured using Euclidean (air-line) distance or along the railroad network, e.g. by measuring the length of the shortest path from one point on the network to another. The choice depends on data availability and research question. We decided to use network-based shortest path distances to measure distances between cases of suicide, because this approach takes the railroad layout and crossings into account.

Using a single, fixed grid introduces a problem known as MAUP (modifiable areal unit problem), i.e. bias introduced by choosing a specific tessellation of the study area or the network [17]. As we also use a grid-based algorithm to estimate the locations of suicide hotspots, we mitigate this problem by averaging over multiple grid definitions with varying origins. For the model simulations, we choose to use a point pattern model that, by definition, is free of MAUP.

We use a density-based clustering approach which (i) can identify spatially connected regions of arbitrary shapes as clusters and (ii) also takes account of the spatial uncertainty of case locations.

*What is a suicide cluster/hotspot?* We aimed to find a definition of a railway suicide hotspot that allows us to identify locations with high case densities while incorporating the spatial uncertainty of case coordinates. As railway suicide hotspot, we define a spatially connected region where the mean density of cases per area is above a threshold. The mean density is calculated separately for each pixel from simulations of a Gaussian noise model created to incorporate the spatial uncertainty of case coordinates. The per-pixel threshold is chosen as the lower 10% quantile from the simulation envelope of the Gaussian noise model. We did not consider background characteristics such as population density (i.e. cases per area unit) for defining clusters, because our interest was on locations with higher overall case numbers, which would be of greatest relevance to suicide prevention efforts.

## 2. Material and methods

### 2.1. Hotspots of railway suicides

*Data on locations of suicides* on the Austrian railroad network from 1998 to 2009 have been obtained from the main Austrian railroad operator ÖBB (*N*=1170).

After removal of cases from Lichtenstein (*N*=4), cases inside trains (*N*=6) or not directly located on the railroad network (*N*=30), 1130 cases of suicide by train impact remain.

Locations are recorded as kilometre-number on the railroad network together with the name of the closest station. This information has been used together with an ÖBB Railway Map [18] and Google Maps (maps.google.com) to obtain the spatial coordinates for each case. We estimate that this procedure has a (Gaussian) measurement uncertainty of approximately 1 km.

*Data on the railroad network* have been obtained from the publicly available Euroglobalmap [19] from 2014. We use the associated metadata to retain only the operational part of the network. We expect the railroad network to remain static over the study period. From 1998 some railroad segments have been closed, but for all suicide cases from 1998 to 2009, a proper network segment exists in the network data from 2014. The total network length is 5916 km (3676 miles).

*Hotspots.* To identify the spatial locations with highest densities of suicides per railroad kilometre, we use box-counting on multiple fixed pixel grids: we divide the study region into a regular grid of quadratic pixels with 1 km side-length and count the number of cases per pixel. To mitigate the introduced discretization error (MAUP) [20], we repeat the procedure 16 times using slightly displaced grids while keeping the pixel size constant (quadrates with 1 km side-length). The displacement is such that the original pixel of 1 km side-length is divided symmetrically into sub-pixels of 250 m side-length.

To take the case location uncertainty of about 1 km into account, we generate 1000 realizations of a Gaussian noise model, i.e. the case locations are displaced along the railroad network using a normally distributed random displacement with *σ*=1 *km*.

For each realization, the box-counting is performed for each of the 16 displaced grids, resulting in a final 250 m wide grid with 16 000 counts per pixel. From this ensemble, we pixel-wisely find the lower 10% quantile of the number of cases. This results in a conservative estimate for the spatial case density distribution.

Finally, we identify the hotspots from the resulting image as eight-connected groups of pixels, i.e. neighbouring pixels with value also above threshold and located sideways, above/below and also diagonally are grouped. Hotspots with 3 pixel minimum border-to-border distance are merged.

We validate the stability of this procedure by using various larger values of grid width. We consistently identify the same hotspots as presented, corresponding to the order of the respective grid width.

### 2.2. Psychiatric institutions and railway suicides

*Data on hospitals* and medical institutions of Austria from 2014 with total bed counts, types of departments and street addresses have been obtained from the Austrian Federal Ministry of Health [21].

The geographical locations of the facilities have been obtained by looking up their street addresses using QGIS [22] with overlay data from Google Maps (maps.google.com), OpenStreetMap (www.openstreetmap.org) or Geoland Basemap (www.basemap.at).

For each institution with a psychiatric department or unit, we calculated the average department size by dividing their total bed counts by the respective number of department types. The resulting psychiatric bed counts are, therefore, proportional to the total number of beds and inversely proportional to the number of department types in the respective facility. This serves as a proxy for psychiatric institution size.

*Testing the possible influence of psychiatric institutions* on railway suicides is done using a threefold approach. First, we test the null hypothesis *H*_0_: *the locations of psychiatric institutions have no influence on locations of railway suicides*, using constrained-realization Monte Carlo (MC) simulations [23] of *H*_0_ (§(c)). Second, we fit an inhomogeneous Poisson process (IPP) to the point pattern of suicides using maximum-likelihood estimation and then generate random samples from the fitted model to estimate the effect size of the influence of psychiatric institutions to nearby suicides (§(d)). Third, we evaluate the model at the identified hotspots to quantify the excess risk associated with the variables at these locations (§(e)).

### 2.3. Testing the null hypothesis

*Testing H_0_* is done by randomly reshuffling the locations of hospitals such that the statistical distribution of psychiatric beds along the railroad network remains close to the original statistical distribution (i.e. the distribution calculated from the original geographical locations). This means, we create random configurations of hospital locations while retaining the property that more institutions are usually located in more densely populated areas.

Let *k* be the bin number, *c*(*k*) the bin centre value (of psychiatric bed density) for bin *k*. Let *n*(*k*) and *n*_0_(*k*) be the frequencies in the randomized and in the original data, respectively.

To measure the deviation of a random configuration (with histogram *n*(*k*)) from the original distribution (with histogram *n*_0_(*k*)), we use a *weighted* *χ*^2^-statistic of the histograms, i.e. we calculate

$$\chi^{2}[n(k)]≑\sum_{k=1}^{\#\;\mathrm{bins}}\frac{{\left(n(k)-n_{0}(k)\right)}^{2}}{n_{0}(k)}\;c(k)$$

with weights *c*(*k*). The weighting ensures that larger psychiatric institutions carry more weight.

To obtain a test statistic allowing us to decide on the rejection of *H*_0_, we fit an inhomogeneous Poisson model using the psychiatric bed density, population density and single versus multitrack railway as explaining variables and the actual suicide locations as dependent variable (the exact procedure is explained in §(d)). As test statistic we use the parameter estimate of the psychiatric bed density. This statistic is calculated for 999 random hospital location configurations and compared with the test statistic obtained for the actual hospital locations.

It is required to select an acceptance/rejection threshold for the *χ*^2^-statistic of the random configurations. A high threshold leads to efficient simulation (fewer rejections), but might also result in inconsistency with the to-be simulated hypothesis *H*_0_. A low threshold ensures this consistency, but is more inefficient to simulate.

After experimenting with different thresholds, we decide on a threshold of 5000, i.e. accept the random configuration if its *χ*^2^≤5000, otherwise we reject it. In order to assess whether this threshold still allows for a consistent simulation of *H*_0_, we determine the sensitivity of the test statistic with regard to *χ*^2^: we group the surrogates into 12 equally sized groups according to their *χ*^2^-value. We then compare the influence of each group on the test statistic. A Kruskal–Wallis test did not reject that the groups result in the same values for the test statistic (*p*=0.62; electronic supplementary material, figure S1). Assuming that the group with smallest *χ*^2^ sufficiently constrains the simulation in order to produce correct simulations of *H*_0_, we conclude that the groups with higher *χ*^2^ values do this as well and that the selected threshold value was adequate.

### 2.4. Fitting an inhomogeneous Poisson model

We model the suicide locations as a spatial point pattern generated by an IPP with a spatially varying log-linear intensity function of the spatial variables:

(a) *Population density*. We obtained publicly available population data of 2006 from Statistics Austria [24], given in absolute numbers of registered residents on a 1 km wide grid. In order to account for some mobility of the population, we calculate a smoothed population map using Gaussian kernel smoothing with a bandwidth of 1.6 km on a 250 m wide destination grid. As fitting the IPP requires the log-density, we first logarithmize the population data and smooth afterwards.
(b) *Psychiatric bed density*. From the approximate number of psychiatric beds per hospital and the hospital locations (§(b)) we create a map of the spatial density of psychiatric beds using Gaussian kernel smoothing with a bandwidth of 0.8 km. This bandwidth choice accounts for the mobility of psychiatric patients which has been reported before [14]. Again, we need the logarithmic psychiatric bed density when fitting the IPP: as the data are zero-inflated, we first offset them by adding one, then logarithmize and finally apply Gaussian kernel smoothing.
(c) *Single track versus multitrack*. We use a binary variable encoding for single versus multitrack segment from the Euroglobalmap data (§(a)). Typically, multitrack segments are associated with a higher amount of railway traffic. The variable can therefore be seen as a proxy for low versus high traffic density.
(d) *Total suicide rate and socio-demographic factors*. We extract two spatial variables as the most important factors from a principal components analysis (PCA) on input data from various sources and spatial resolutions. These are: (i) mean yearly gross income of employees (in EUR, from 2003, on a district level [25]), (ii) fraction of population older than 15 years with low (no completed secondary education), medium (completed secondary) and with high (completed tertiary) eduction (2011, on a community level [26]), (iii) fraction of male/female population, (iv) fraction of young (age ≤19 years), adult (20 ≤ age ≤ 64) and elderly (age ≥65) population, (v) fraction of persons without Austrian or EU citizenship, (vi) fraction of unemployed persons (iii)–(vi) on a 10 km grid, from 2008 [27]), (vii) the number of suicides in the years 2001–2008 per mean population in the same time span (district level).

To perform the PCA, we sample these variables—together with the population density from above—at 20 000 random points on the railroad network. On these data we perform a population-weighted, centred PCA. The first two components are then used as variables in the analyses.

*Fitting the IPP* is done using the practical maximum-likelihood approach of Baddeley *et al.* [28]. We adapt the procedure to work with a network tessellation instead of a two-dimensional one. The procedure works as follows:

1. Displace the original cases randomly (normally distributed with *σ*=1 *km*) along the railroad network to take the measurement uncertainty of the case locations (§(a)) into account. These are the *data points*.
2. Select 20 000 random *dummy points* on the network. The higher the number of dummy points, the better the accuracy of the maximum-likelihood estimates. This number has been chosen high enough to give stable results when repeating this procedure.
3. Compute the network weights *w*_*i*_ for data and dummy points, i.e. the length of its network share (*i* numbering the points).
  Data and dummy points together define a (non-unique) network tessellation, i.e. each point gets assigned its respective share of the network. The tessellating procedure we use is similar to the Voronoi tessellation of a network [29], p. 84, but we also handle *critical nodes*. A critical node is a point that prohibits mutually exclusive Voronoi segments, i.e. leads to overlapping Voronoi segments. We handle this by dividing the lengths of overlapping segments to their adjacent points proportionally to the inverse shortest-path distance between this overlapping segment and its respective adjacent point.
4. Set the dependent variable *y*_*i*_=1/*w*_*i*_ for each data point and *y*_*i*_=0 for each dummy point.
5. Find the vector of explaining variables *v*_*j*_(*x*_*i*_) at each data and dummy point (*j* numbering the variables).
6. Fit a Poisson generalized linear model to find the coefficient estimates *θ*_*j*_ using variables *v*_*j*_(*x*_*i*_), weights *w*_*i*_ and responses *y*_*i*_.

*Incorporating case location uncertainty*. To account for the case location uncertainty, we simulate a Gaussian noise model on the network with *σ*≈1 km 39 times and each time perform the IPP fit described above. The resulting mean coefficient estimates are then used to create 199 simulated realizations of the IPP: we use a regular grid with 250 m wide pixels to simulate the fitted model. From the mean coefficient estimates, the explaining variables evaluated at pixel centres, and the network length per pixel, we create a two-dimensional IPP intensity image. Each realization creates 1130 cases distributed on the map proportional to this intensity image.

As the outcomes of this simulation procedure strongly depend on the grid width (i.e. smaller is more accurate), we use a *Z*-test based on the simulation variance to compare the simulation input coefficients with their corresponding mean simulation coefficients.

*Goodness of fit*. The goodness of fit is estimated by calculating the pair distribution function together with simulation envelopes from the simulated model. The pair distribution function, also called pair correlation function, is the histogram of all pairwise distances of the points of a point pattern, normalized with respect to complete spatial randomness (CSR) [30]. We defined CSR as a homogeneous Poisson process on the railroad network, which has been estimated by simulation of 199 realizations. Note that the point-wise 95%-simulation envelope for CSR was at any point within 1±0.5 (electronic supplementary material, figure S2). The distances were measured as shortest-path distances on the railroad network.

*Temporal pair correlation*. We also take a look at the purely temporal pair correlation functions, i.e. not considering spatial locations of suicides and baselining to a homogeneous Poisson process in time. There is no temporal clustering visible, neither on short nor on long time scales (electronic supplementary material, figure S3).

### 2.5. Evaluating the model at hotspots

Above (§(a)) we identified (groups of) pixels as hotspots. We calculate the excess risk ER_*j*_ associated with a variable *j* at a given hotspot as the *mean value* of excess risk across the pixels of the respective hotspot.

To calculate the excess risk er_*j*_(*x*_*i*_) associated with variable *j* at pixel *x*_*j*_, we use the log-linear model to find that the intensity $\lambda {\left(x_{i})=\prod_{j}(v_{j}(x_{i})\right)}^{\theta_{j}}≑\prod_{j}{\text{er}}_{j}(x_{i})$ with ${\text{er}}_{j}(x_{i})≑{\left(v_{j}(x_{i})\right)}^{\theta_{j}}$.

### 2.6. Software

The analyses have been performed using Matlab [31] with Statistics and Machine Learning Toolbox. Data preparation and cleaning have been done using R [32], rgdal [33] and GDAL [34].

## 3. Results

### 3.1. Hotspots

Table 1 shows the 15 identified hotspots on the Austrian railway network. For each hotspot we provide the number of pixels included, the approximate number of suicide cases, and the distance to the closest psychiatric institution. We also show the evaluations of the population and railway variables for each hotspot, i.e. the excess risks for population density, psychiatric bed density, multitrack versus single-track railway and socio-economic components. We used the first two components of a PCA, explaining 89.8% of the variance, with component 1 loading high on high education and component 2 loading high on total suicide rates.

**Table 1.** Table of identified hotspots, sorted by the approximate number of cases per hotspot. Distances are given in kilometres air line. Coordinates are of maximum density of given hotspot (in EPSG:3035). Pixels are quadrates with side lengths of 250 m. Excess risk is calculated from the fitted model evaluated at the hotspot pixels as described in the main text (see §(e)). Mödling is listed twice as closest main station, because of two different hotspots. Comp1 is principal component 1 and comp2 is principal component 2.

				closest main station		excess risk				
no.	area (no. of pixel)	approx. no. of cases	closest psychiatr. inst. (km)	name	distance (km)	pop.	psy.	multitrack	comp1	comp2
1	39	26–32	3.0	Mödling	5.2	8.2	1.0	1.2	1.4	0.89
2	27	23–28	0.51	Salzburg Hauptbahnhof	2.3	8.5	2.9	1.2	1.3	0.98
3	36	19–27	3.5	Klagenfurt Hauptbahnhof	4.6	4.2	1.0	1.2	1.4	1.2
4	19	10–16	2.3	Hall in Tirol	2.0	4.4	1.5	1.2	1.2	0.99
5	12	6–11	7.1	Dornbirn	0.64	5.3	1.0	1.2	1.1	0.99
6	15	5–11	0.73	Wels Hauptbahnhof	0.045	6.8	2.0	1.4	1.1	0.89
7	11	4–10	0.28	Graz Hauptbahnhof	4.0	7.8	2.9	1.2	1.4	1.2
8	10	4–9	6.6	Salzburg Hauptbahnhof	5.9	5.1	1.0	1.2	1.3	0.99
9	11	4–9	1.0	Linz Hauptbahnhof	0.18	8.5	1.6	1.2	1.2	1.1
10	11	3–8	12	Leobersdorf	5.6	3.7	1.0	1.2	0.91	0.93
11	7	2–7	12	Bischofshofen	0.78	3.2	1.0	1.2	0.92	1.2
12	6	2–7	4.0	Mödling	2.9	3.7	1.0	1.2	1.2	0.82
13	4	1–5	1.8	Amstetten	5.8	3.7	1.1	1.0	1.0	1.1
14	4	1–5	1.8	Baden	1.7	4.5	1.1	1.3	1.3	0.99
15	4	1–5	0.96	Wien Meidling	1.5	14	1.8	1.2	1.1	0.94

**Figure 1.**
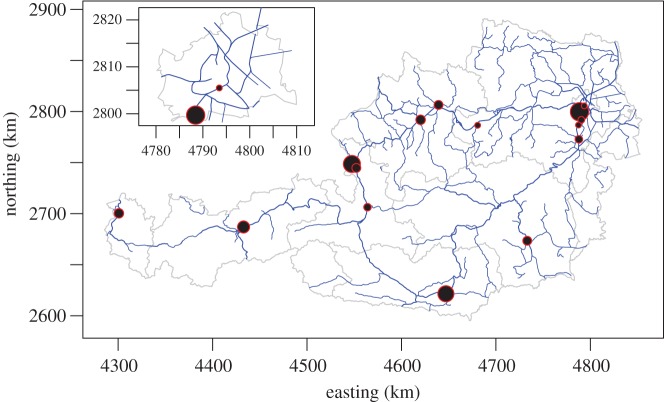
Locations of the hotspots from table 1. The dot area is proportional to the approximate number of railway suicide cases (under a Gaussian noise model) at the identified hotspot. Blue lines: railroad network, thicker lines: multitrack. Grey lines: national boundary of Austria and boundaries of federal states. The inset shows a closeup of the capital city of Vienna. This figure has been created using R [32], rgdal [33] and GDAL [34].

Figure 1 shows their locations represented as dots. The size of the dots corresponds to the approximate number of railway suicide cases.

### 3.2. Inhomogeneous Poisson model

Table 2 shows the maximum-likelihood estimates (means and standard deviations) based on 199 simulations. The *Z*-scores of the simulation input parameters in relation to the statistical distribution resulting from the simulated model indicate that the discretization error of the simulation is negligible, i.e. the grid width is sufficiently small.

**Table 2.** Simulated model. Mean and standard deviation of the maximum-likelihood estimates of 199 Monte Carlo simulations of the fitted model and comparison with simulation inputs.

	maximum-likelihood estimates		comparison to simulation inputs	
explaining variable	mean	standard deviation	*Z*	*p*-value
intercept	−3.77	0.100	−0.76	0.44
psy *h*=0.8 km	17.5	2.8	−0.20	0.84
multitrack line	1.54	0.076	−0.47	0.64
pop *h*=1.6 km	4.85	0.36	1.0	0.31
comp1	0.295	0.081	−0.28	0.78
comp2	0.569	0.14	0.13	0.90

**Figure 2.**
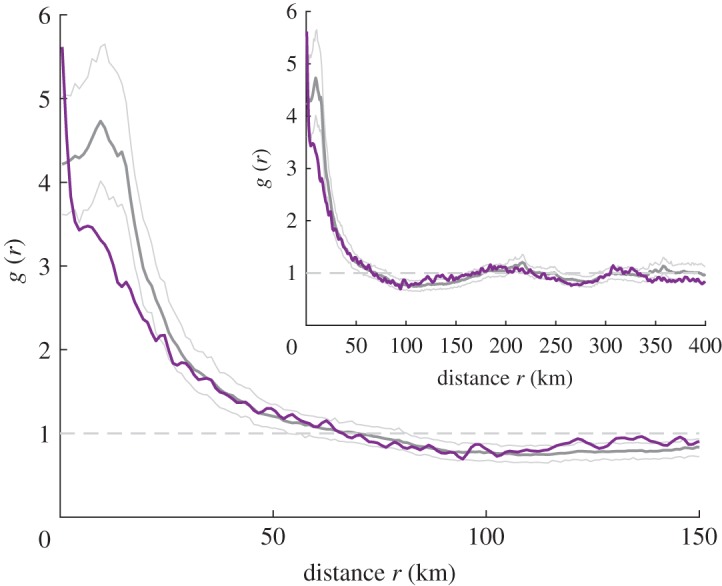
Pair distribution functions *g*(*r*). The dashed grey line (*g*(*r*)≡1) represents complete spatial randomness (CSR). The dark grey line is *g*(*r*) of 199 simulation realizations of the fitted model and the light grey lines its 95% point-wise simulation envelope. The purple line shows the pair distribution function of the original suicides point pattern. The main plot shows a close-up for distances up to 150 km, whereas the inset shows *g*(*r*) for up to 400 km. The network diameter is 761 km which is the maximum possible pairwise distance. This figure has been created using Matlab [31].

Figure 2 compares the pair distributions from the original point pattern with pair distribution of the simulated model. The figure illustrates (i) the clear difference of the suicide pattern from CSR, (ii) that the suicide point pattern seems generally well represented by the fitted model, and (iii) a possible non-modelled over-clustering at very small distances of up to a few kilometres.

### 3.3. Psychiatric institutions

We create surrogate data from the null hypothesis using constrained-realization MC 999 times and calculate the test statistic. The result is approximately normally distributed with *μ*=−0.58±0.02 and *σ*=0.57±0.02. The test statistic for the original data is 0.74 which leads to rejection of *H*_0_ with *p*<0.011.

## 4. Discussion

### 4.1. Main findings

In this study, we identified several hotspots of railway suicides along the railway network. The 15 hotspots identified in this study comprised between 111 and 190 railway suicides, which corresponds to 9.8%–16.8% of all railway suicides in the observation period. The specific locations were spread across the entire country, with eight of nine federal states having at least one hotspot, covering approximately 54 km of the railway track in total (0.9% of the entire track length). A previous Dutch study on railway suicides similarly found that 16% of all railway suicides occurred on just 33 km of the Dutch trail track, which was about 1.2% of the total track length [7].

In order to model railway suicides we assumed a smoothened IPP. We tested the influence of psychiatric bed density, single versus multitrack railway; as well as population density on railway suicides. We controlled the model for two principal components of a wide range of spatial socio-economic factors, including population income level, education, proportion of males and females, age groups, proportion of residents without EU citizenship, unemployment rates, as well as total suicide rates. The two components used loaded high on education (component 1) and total suicide rates (component 2), as well as moderately on income level (component 2).

The findings indicated that railway suicides were associated with proximity of psychiatric institutions in walking distance to the railway network. The closer and higher the number of psychiatric beds in the proximity of the railway network was, the more suicides occurred on the railway track. An evaluation of the model for the specific hotspots indicated that in 5 of 15 identified hotspots, psychiatric institutions were not more than 1 km away from the railway. The excess risk associated with proximity to psychiatric institutions was larger than 1.5 in 6 of 15 hotspots, and ranged from 1.5 to 2.9 depending on the hotspot, indicating a small to medium effect size. A validation of the model with a randomization test which shuffled psychiatric institutions on the railway network taking account of the population density in the specific areas, confirmed this result. Several previous studies have described that proximity of railway tracks to psychiatric institutions may increase the number of railway suicides in that area [7,9,12]. However, none of these studies have applied the present three-step methodological approach. In a Dutch study, it turned out that all locations on the network with the highest frequency of suicides per kilometre had psychiatric institutions within 800 m walking distance [7]. Similarly, a study from Germany indicated that 75% of the German railway locations with highest suicide density had psychiatric institutions nearby [12]. These studies were descriptive, and did not control for any demographic or railway factors. A survey study from Brisbane, Australia indicated that 48% of railway suicides occurred close to a psychiatric institution, and 57% of decedents had been treated for mental health problems [9].

Also consistent with earlier research, local population density was associated with railway suicides along the railway track [4]. In the UK, railway suicides were typically found around major cities with high population density [11]. By contrast, the Dutch evaluation of railway suicides found similar rates of train suicides in three regions with different population densities, with a lower rate in the most densely populated region [7]. The authors of this study concluded that tracks may be less accessible in Dutch areas with high population density. However, similar to the other previous analyses, this analysis did not take small-area variations in population densities and railway suicides into account. In the present analysis, the individual evaluation of 15 hotspots indicated that population density was relevant to all of these locations, with excess risks ranging from 4.2 to 14, which corresponds to medium to large effect sizes.

We also found an independent association between multitrack lines on the Austrian railway network and railway suicides, with excess risks ranging from 1.0 to 1.4 for the individual hotspots. Previous studies analysed if the availability of trains, or passenger numbers were associated with railway suicide and showed mixed results [7]. The previously mentioned analysis of Dutch railway suicides did not identify a correlation between railway suicide and railway density or intensity of railway transportation as indicated by the variations in railway suicides across four areas with different rail density [7]. By contrast, railway suicides in England and Wales were correlated with the growth of the railway system over time [35].

Total spatial suicide rates, which had a very high loading in the second principal component used in the analysis, were associated with railway suicides at some but not all of the identified hotspots. Excess risks were negative or positive, depending on the individual hotspot, and overall sizes of risk estimates were small. A previous study identified decreasing total suicide rates but increasing railway suicides in a 10-year period of 1991 to 2000 in Germany [36]. Similarly, Dutch railway suicides did not show the same longitudinal time patterns as total suicide rates [7]. The small and inconsistent relationship of the spatial distributions of total suicides and railway suicides in the present study highlights that the impact of total suicides on railway suicides is low, inconsistent, and, if there is any at all, only relevant at selected hotspots.

Spatial socio-economic factors, particularly high level of education, which loaded strongly in the first principal component used in this analysis, were related to railway suicide at several hotspots, but again, the associations were not consistent. At 13 of the 15 hotspots, there was a small direct association between high level of education and railway suicides, suggesting that areas with higher level of education of the population are more affected by railway suicide. Interestingly, these 13 hotspots were all located close to urban centres, whereas the two hotspots with divergent findings were located in rural areas. Education may reflect an uncontrolled effect of urbanity. We are not aware of any study assessing spatial associations of socio-economic variables with railway suicides, but a meta-analysis investigating the association of spatial socio-economic conditions with total suicide rates indicated substantial heterogeneity of findings, with 30% of studies with significant findings reporting direct associations and 70% reporting inverse associations.

A crucial step in any analysis on potential clustering is the definition of a cluster. A recent review of studies on suicide clustering identified that only a third of 82 included studies defined what they meant by clusters, and only 27 studies provided data as to how many suicides may have occurred in a cluster [37]. In this study, we defined railway suicide hotspots (or clusters) as a spatially connected region where the median density of cases per area was above a threshold calculated from simulations of a Gaussian noise model created to incorporate the spatial uncertainty of case coordinates. The threshold was chosen as the lower 10 per cent quantile from the simulation envelope of the Gaussian noise model. This is different from the Centers for Disease Control’s definition of suicide clusters as ‘a group of suicides that occur closer together in time and space than would normally be expected in a given community’ [38]. We neither considered background characteristics such as population density (i.e. cases per area unit), nor did we use a time component in our present definition, because our interest was on locations with the overall highest case densities, which would be of greatest relevance to suicide prevention efforts on the railway network.

The present model, while having a good model fit, indicated some potential over-clustering of railway suicides at small distances of up to 20 km. This unexplained over-clustering at small distances may be due to specific environmental characteristics not controlled for in the model or due to imitative behaviour, which has been shown to be typically limited to areas of up to 50 km radius maximum [39]. Imitative behaviour can occur due to social contagion effects [39]. For example, media reporting on a railway suicide may trigger further suicides in the same location, which may again increase media reporting, resulting in a vicious circle of heightened media attention and more suicides [15]. Contagion effects typically show not only a spatial clustering but also a time clustering, i.e. their duration is limited. A review of studies on suicide clustering indicated that the median duration of clusters was 11 months, with a range of 0–24 months [40]. Owing to the absence of any visible time clustering in this study, our analysis does rather point to structural, more permanent factors that impact on the clustering of railway suicides at small distances in specific areas. These factors may either be population- or area-related.

### 4.2. Implications for research and prevention

This study has several implications for future research in the area of suicide clustering on networks such as on railway tracks. It shows that standard methods are applicable in this area which has been characterized by a lack of standardized statistical approaches to data analysis. Future studies may use this study as a basis for further in-depth analysis of spatial distributions of suicides on networks.

There are several important implications for suicide prevention. First, this study shows that a significant proportion of suicides occurs on a very small proportion of the network. Barriers and other structural measures may be applicable to prevent railway suicides at these hotspots [4]. In this context, particularly psychiatric institutions need to be structurally separated from railway tracks. However, this study also highlights that not all locations with relatively high railway suicide densities show the same characteristics. While population density seems to play a major role at several locations on the Austrian railway network, vicinity to psychiatric institutions seems to play a stronger role at other hotspots. A careful site inspection of the identified hotspots seems warranted in order to assess specific conditions and develop adequate prevention techniques tailored to the specific hotspots. Beside fences and spatial separation, media collaborations to prevent imitative effects, as well as speed control have been proposed as preventive measures in the literature [4]. Further, front modifications of trains as well as wheel and rail designs may contribute to the prevention of lethal consequences of train–person collisions [4]. Most of these measures have not been evaluated with regard to their effectiveness for suicide prevention. In Switzerland, barriers on bridges have been shown to significantly reduce suicidal behaviour [41]. Suicides at nearby bridges within 5 min walking distance did not increase subsequently. Similarly, a minimal structural intervention of installing guard rails at hospital windows significantly reduced the number of patients dying by jumping out of windows [42]. A meta-analysis of structural interventions at suicide hotspots confirmed that there was an overall preventive effect of these interventions on suicides by jumping [43].

### 4.3. Strengths and limitations

This study has several strengths. With regard to the identification of hotspots, using a single, fixed grid introduces a problem known as MAUP, i.e. a bias introduced by choosing a specific tessellation of the area or the network. Therefore, during the hotspot estimation, we averaged over multiple grid definitions with varying origins. For the model simulations, we used a point pattern model that, by definition, is free of MAUP [17]. In recent studies on suicide clustering [37,39,44], statistical software such as SatScan has been used, which basically works by moving windows of elliptical shape and varying size over the study region and identifies clusters using a statistical criterion [20]. Because the railway represents a network and suicides can only occur on that network, the present density-based clustering approach was superior in that (i) it allowed the identification of spatially connected regions of arbitrary shapes as clusters and (ii) it also allowed to take into account the spatial uncertainty of case locations.

Also the modelling technique used here has several strengths. While earlier studies typically assessed correlations between parameters such as population density and railway suicides on an aggregate level over time, we were able to investigate spatial variations of densities across the whole length of the railway track. The present statistical modelling allowed us to take probable measurement errors regarding the specific locations of individual railway suicides into account. The adjustment of the model for socio-demographic variables as well as for the total suicide rate is a strength, as well as the validation of the model using randomization tests.

Despite many strengths, this study also has some limitations. Not all socio-demographic variables were available at the level of communities; therefore, we needed to use some district data, which may introduce bias [45]. Further, the density of psychiatric beds was an approximate measure based on the average number of beds per department type. This measure reflects the average department type size of a given institution. Actual psychiatric bed numbers may deviate from the present densities. Future studies should use more accurate data for the size of psychiatric institutions. However, also average bed numbers per department type as calculated here might be related to suicide hotspots independent of the specific size of the psychiatric departments.

Importantly, no information on socio-demographic and morbidity-related characteristics of the suicide decedents was available. We do not know if the suicide decedents had a mental illness or if they were patients at the respective institutions. Finally, the measurement of distances between the rail track and psychiatric institutions in terms of Euclidian distance (air-line) may not be entirely accurate, because this assumes that individuals can move freely in all directions. Future studies may use other network information, e.g. the sidewalk structures, to determine distances between objects and the railway network.

Finally, the model might have been stronger if some measure of existing suicide barriers (e.g. walls and fences) could have been added. For example, some psychiatric institutions might be physically separated from tracks. These barriers may effectively reduce the risk of suicide at these locations. Unfortunately, data on existing barriers were not available for this study.

## 5. Conclusion

For the first time, we show here that spatial clustering of suicide hotspots can be understood with unprecedented precision through models for point processes. This has immediate consequences for suicide prevention. The study indicates that a considerable number of railway suicides occur on a very short fraction of the total network. Vicinity to psychiatric institutions, population densities, as well as multitrack parts of the network are particularly relevant and warrant attention in tailored prevention efforts. Spatial socio-economic factors and total suicide rates are not consistently associated with railway suicides.

## Supplementary Material

Supplementary Figures S1-S3

## Data accessibility

Owing to the sensitive nature of the data used in this analysis, we received an exception from the Journal’s data sharing policy regarding the sharing of primary data. We requested this waiver particularly for our primary data on the specific locations and dates of individual railway suicides. These data are sensitive in nature. They allow one to identify individual cases of suicides. In the paper, we do not provide specific locations for individual acts and only approximate locations for the spots with the highest number of cases. Individuals interested in the data can contact the Austrian Federal Railways (ÖBB) who is maintaining the dataset. In order to allow other researchers to apply and test our approach, we publish the code used in the three analytic sections of the paper, along with surrogate data, and with supporting documents at: https://github.com/mstrauss/hotspots/. This includes the code for all three parts of the analysis, i.e.

1. the identification of hotspots, taking account of the case location uncertainty;
2. the simulation of the null hypothesis (i.e. spatial vicinity to psychiatric institutions is not associated with railway suicides); and
3. the evaluation of the model for the individual hotspots.

## References

[1] World Health Organisation. 2014 *Preventing suicide. A global imperative*. Geneva, Switzerland: WHO.

[2] Bundesministerium für Gesundheit. 2015 *SUPRA Suizidprävention Suizid und Suizidprävention in Österreich*. Vienna, Austria: BMG.

[3] Beskow J, Thorson J, Öström M 1994 National suicide prevention programme and railway suicide. *Soc. Sci. Med.* 38, 447–451. (doi:10.1016/0277-9536(94)90446-4) 8153750 10.1016/0277-9536(94)90446-4

[4] Røadbo H, Svedung I, Andersson R 2008 Suicide prevention in railway systems: application of a barrier approach. *Saf. Sci.* 46, 729–737. (doi:10.1016/j.ssci.2006.12.003)

[5] Symonds R 1994 Psychiatric and preventative aspects of rail fatalities. *Soc. Sci. Med.* 38, 431–435. (doi:10.1016/0277-9536(94)90443-X) 8153747 10.1016/0277-9536(94)90443-x

[6] Ratnayake R, Links PS, Eynan R 2007 Suicidal behaviour on subway systems: a review of the epidemiology. *J. Urban Health* 84, 766–781. (doi:10.1007/s11524-007-9211-5) 17828459 10.1007/s11524-007-9211-5 PMC2232036

[7] van Houwelingen CA, Kerkhof AJ, Beersma DG 2010 Train suicides in the Netherlands. *J. Affect. Disord.* 127, 281–286. (doi:10.1016/j.jad.2010.06.005) 20580436 10.1016/j.jad.2010.06.005

[8] Deisenhammer EA, Kemmler G, De Col C, Fleischhacker WW, Hinterhuber H 1997 Eisenbahnsuizide und -suizidversuche in Österreich von 1990–1994. *Erweiterung der Hypothese medialer Vermittlung suizidalen Verhaltens. Nervenarzt* 68, 67–73. 10.1007/s001150050098 9132623

[9] Emmerson B, Cantor C 1993 Train suicides in Brisbane, Australia, 1980–1986. *Crisis* 14, 90–94. 8252930

[10] van Houwelingen CA, Kerkhof AJ 2008 Mental healthcare status and psychiatric diagnoses of train suicides. *J. Affect. Disord.* 107, 281–284. (doi:10.1016/j.jad.2007.08.024) 17913238 10.1016/j.jad.2007.08.024

[11] Abbott R, Young S, Grant G, Goward P, Seager P, Ludlow J 2003 Railway suicide: an investigation of individual and organizational consequences. A report of the SOVRN project. Doncaster and South Humber Healthcare NHS Trust.

[12] Erazo N, Baumert J, Ladwig KH 2004 Regionale und örtliche Verteilungsmuster von Bahnsuiziden. *Nervenarzt* 75, 1099–1106. (doi:10.1007/s00115-004-1703-x) 15549217 10.1007/s00115-004-1703-x

[13] van Houwelingen C, Baumert J, Kerkhof A, Beersma D, Ladwig KH 2013 Train suicide mortality and availability of trains: a tale of two countries. *Psychiatry Res.* 209, 466–470. (doi:10.1016/j.psychres.2012.12.026) 23380544 10.1016/j.psychres.2012.12.026

[14] van Houwelingen CA 2011 Studies into train suicide. PhD thesis, Vrije Universiteit, Amsterdam, The Netherlands.

[15] Niederkrotenthaler T, Sonneck G, Dervic K, Nader IW, Voracek M, Kapusta ND, Etzersdorfer E, Mittendorfer-Rutz E, Dorner T 2012 Predictors of suicide and suicide attempt in subway stations: a population-based ecological study. *J. Urban Health* 89, 339–353. (doi:10.1007/s11524-011-9656-4) 22318375 10.1007/s11524-011-9656-4 PMC3324611

[16] Ladwig KH, Kunrath S, Lukaschek K, Baumert J 2012 The railway suicide death of a famous German football player: impact on the subsequent frequency of railway suicide acts in Germany. *J. Affect. Disord.* 136, 194–198. (doi:10.1016/j.jad.2011.09.044) 22036798 10.1016/j.jad.2011.09.044

[17] Wong D 2008 The modifiable areal unit problem (MAUP). In *The SAGE handbook of spatial analysis* (eds AS Fotheringham, PA Rogerson), pp. 105–123. London, UK: Sage.

[18] ÖBB Infra. 2010 *Eisenbahnatlas Österreich*. Vienna, Austria: Schweers+Wall.

[19] EuroGlobalMap 1:1 million scale topographic dataset. See http://www.eurogeographics.org/products-and-services/euroglobalmap (retrieved 29 April 2014).

[20] Lawson A 2006 *Statistical methods in spatial epidemiology*. Chichester, UK: Wiley.

[21] Bundesministerium für Gesundheit. Krankenanstaltenverzeichnis (not available any more). See http://www.bmg.gv.at (accessed 1 February 2015).

[22] QGIS Development Team. 2016 QGIS Geographic Information System [Computer Software]. Version 2.10. See https://www.qgis.org/ (Open Source Geospatial Foundation, 2016).

[23] Theiler J, Prichard D 1996 Constrained realization Monte-Carlo method for hypothesis testing. *Phys. D Nonlinear Phenom.* 94, 221–235. (doi:10.1016/0167-2789(96)00050-4)

[24] Statistik Austria. 2015 Bevölkerungsstand 2006—ETRS-LAEA 1 km. See http://www.statistik.at/web_de/klassifikationen/regionale_gliederungen/regionalstatistische_rastereinheiten/index.html (accessed 10 August 2015).

[25] Wirtschaftskammer Österreich. Arbeitnehmereinkommen 2003. See http://wko.at/statistik/bezirksdaten/aneinkommen.pdf (accessed 5 November 2016).

[26] Statistik Austria. Gemeindeergebnisse der Abgestimmten Erwerbsstatistik und Arbeitsstättenzählung ab 2011. See http://data.statistik.gv.at/web/meta.jsp?dataset=OGDEXT_AEST_GEMTAB_1 (accessed 5 November 2016).

[27] Paket Abgestimmte Erwerbsstatistik 2008—ETRS-LAEA 10 km. See http://statistik.gv.at/wcm/idc/idcplg?IdcService=GET_NATIVE_FILE&RevisionSelectionMethod=LatestReleased&dDocName=065894 (accessed 5 November 2016).

[28] Baddeley A, Turner R 2000 Practical maximum pseudolikelihood for spatial point patterns. *Aust. N. Z. J. Stat.* 42, 283–315. (doi:10.1111/1467-842X.00128)

[29] Okabe A, Sugihara K 2012 *Spatial analysis along networks: statistical and computational methods*. New York, NY: John Wiley & Sons.

[30] Diggle P 1983 *Statistical analysis of spatial point patterns*. London, UK: Academic Press.

[31] MATLAB [Computer Software]. Version 9.0.0.341360 (R2016a). See https://www.mathworks.com/ (The MathWorks, Inc., 2016).

[32] R Core Team. 2016 R: a language and environment for statistical computing [Computer Software]. Version 3.2.4 (2016-03-10). See https://www.R-project.org/ (R Foundation for Statistical Computing, 2016).

[33] Bivand R, Keitt T, Rowlingson B 2016 rgdal: Bindings for the Geospatial Data Abstraction Library [Computer Software]. R package version 1.1-10. See https://CRAN.R- project.org/package=rgdal (2016).

[34] GDAL Development Team. 2016 GDAL—Geospatial Data Abstraction Library [Computer Software]. Version 1.11.5. See http://www.gdal.org (Open Source Geospatial Foundation, 2016).

[35] Clarke RV, Poyner B 1994 Preventing suicide on the London Underground. *Soc. Sci. Med.* 38, 443–446. (doi:10.1016/0277-9536(94)90445-6) 8153749 10.1016/0277-9536(94)90445-6

[36] Baumert J, Erazo N, Ladwig KH 2006 Ten-year incidence and time trends of railway suicides in Germany from 1991–2000. *Eur. J. Public Health* 16, 173–178. (doi:10.1093/eurpub/cki060) 16093307 10.1093/eurpub/cki060

[37] Niedzwiedz C, Haw C, Hawton K, Platt S 2014 The definition and epidemiology of clusters of suicidal behavior: a systematic review. *Suicide Life-Threat. Behav.* 44, 569–581. (doi:10.1111/sltb.12091) 24702173 10.1111/sltb.12091

[38] Centers for Disease Control (CDC). 1988 CDC recommendations for a community plan for the prevention and containment of suicide clusters. *Morbid. Mortal. Week. Rep.* 37, 1–12. 2841564

[39] Jones P *et al.* 2013 Identifying probable suicide clusters in Wales using national mortality data. *PLoS ONE* 8, e71713 (doi:10.1371/journal.pone.0071713) 24015189 10.1371/journal.pone.0071713 PMC3756004

[40] Larkin GL, Beautrais AL 2012 *Geospatial mapping of suicide clusters*. The National Centre of Mental Health Research, Information and Workforce Development.

[41] Reisch T, Michel K 2005 Securing a suicide hot spot: effects of a safety net at the Bern Muenster Terrace. *Suicide Life-Threat. Behav.* 35, 460–467. (doi:10.1521/suli.2005.35.4.460) 16178698 10.1521/suli.2005.35.4.460

[42] Mohl A, Stulz N, Martin A, Eigenmann F, Hepp U, Hüsler J, Beer JH 2012 The ‘Suicide Guard Rail’: a minimal structural intervention in hospitals reduces suicide jumps. *BMC Res. Notes* 5, 408 (doi:10.1186/1756-0500-5-408) 22862804 10.1186/1756-0500-5-408 PMC3439295

[43] Pirkis J, Spittal MJ, Cox G, Robinson J, Cheung YTD, Studdert D 2013 The effectiveness of structural interventions at suicide hotspots: a meta-analysis. *Int. J. Epidemiol.* 42, 541–548. (doi:10.1093/ije/dyt021) 23505253 10.1093/ije/dyt021

[44] Cheung YTD, Spittal MJ, Williamson MK, Tung SJ, Pirkis J 2014 Predictors of suicides occurring within suicide clusters in Australia, 2004–2008. *Soc. Sci. Med.* 118, 135–142. (doi:10.1016/j.socscimed.2014.08.005) 25112568 10.1016/j.socscimed.2014.08.005

[45] Rehkopf DH, Buka SL 2006 The association between suicide and the socio-economic characteristics of geographical areas: a systematic review. *Psychol. Med.* 36, 145–157. (doi:10.1017/S003329170500588X) 16420711 10.1017/S003329170500588X

