# Supplementary material for: Suicides on the Austrian railway network: hotspot analysis and effect of proximity to psychiatric institutions
Source: R Soc Open Sci. 2017 Mar 8;4(3):160711. doi: 10.1098/rsos.160711 (PMC5383816; doi:10.1098/rsos.160711)
Supplement: Supplementary Figures S1-S3 [file rsos160711supp1.pdf]

Suicides on the Austrian railway network: hotspot analysis and effect of proximity to psychiatric institutions

Supplementary figures

Markus J. Strauss[1], Peter Klimek[2], Gernot Sonneck[3] and Thomas Niederkrotenthaler[1]

[1] Suicide Research Unit, Department of Social and Preventive Medicine, Center for Public Health, Medical University of Vienna, Kinderspitalgasse 15, 1090 Vienna, Austria,
[2] Section for Science of Complex Systems, Medical University of Vienna, Spitalgasse 23, 1090 Vienna, Austria,
[3] Crisis Intervention Centre, Lazarettgasse 14A, 1090 Vienna, Austria.

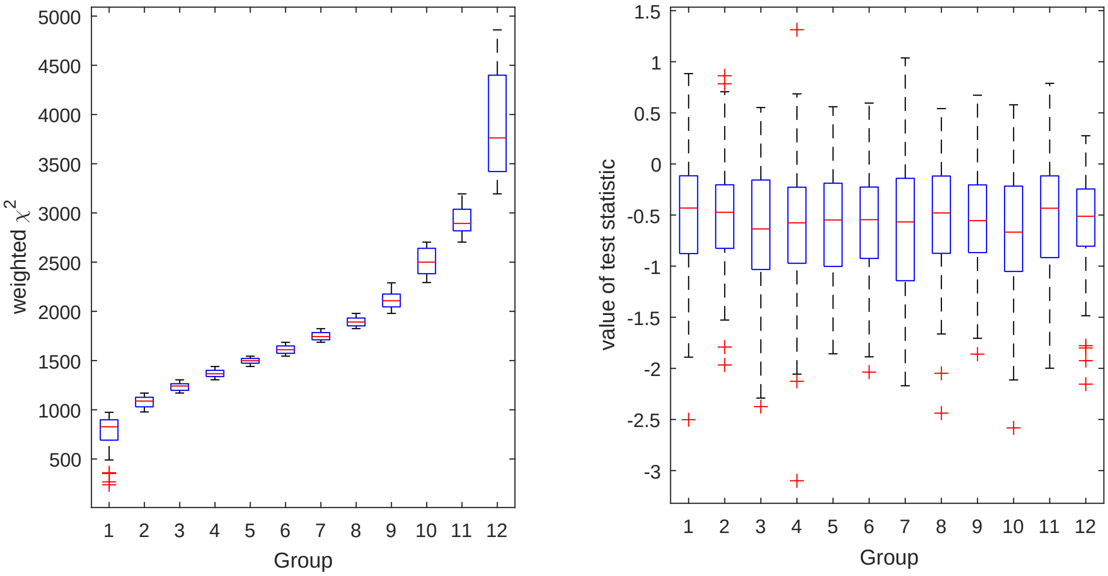

Supplementary Figure S1: Testing the sensitivity of χ^2 on the test statistic. Left: Shows boxplots of the χ^2-values for the equally-sized 12 groups. As visualized, the χ^2-values vary between ≈ 200–1000 (group 1) and ≈ 3.300–4.900 (group 12). Right: Boxplots of the test statistic (regression coefficient of psychiatric bed density) for the same 12 groups. The groups show no variation in the test statistic.

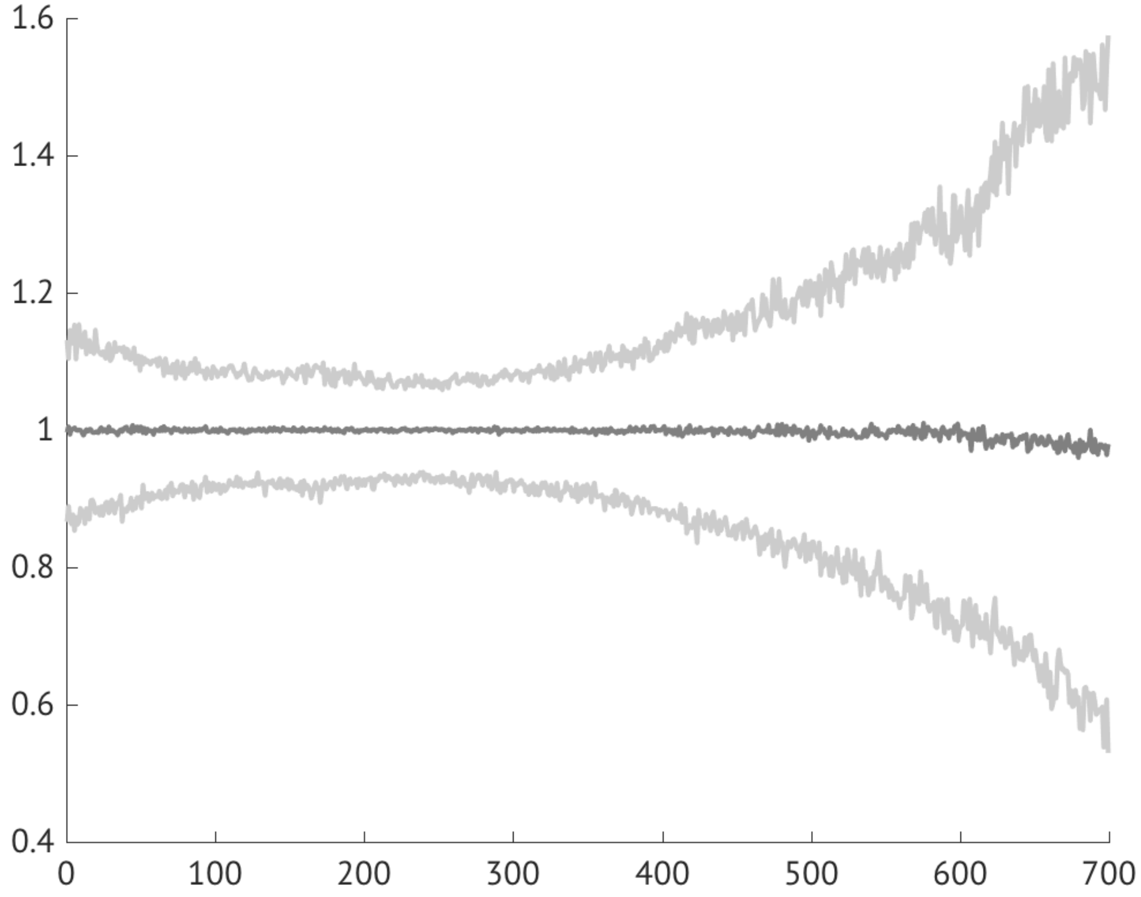

Supplementary Figure S2: Pair distribution function (dark grey line) of the baseline homogeneous Poisson process on the railway network. The abscissa shows the distance in kilometers (the network diameter is 761 km). The light grey lines are the point-wise 95 % simulation envelopes calculated from 1000 realizations of the baseline Poisson process with a rate of 1130 cases per 5.916 km $\approx$ 0.19 cases per km.

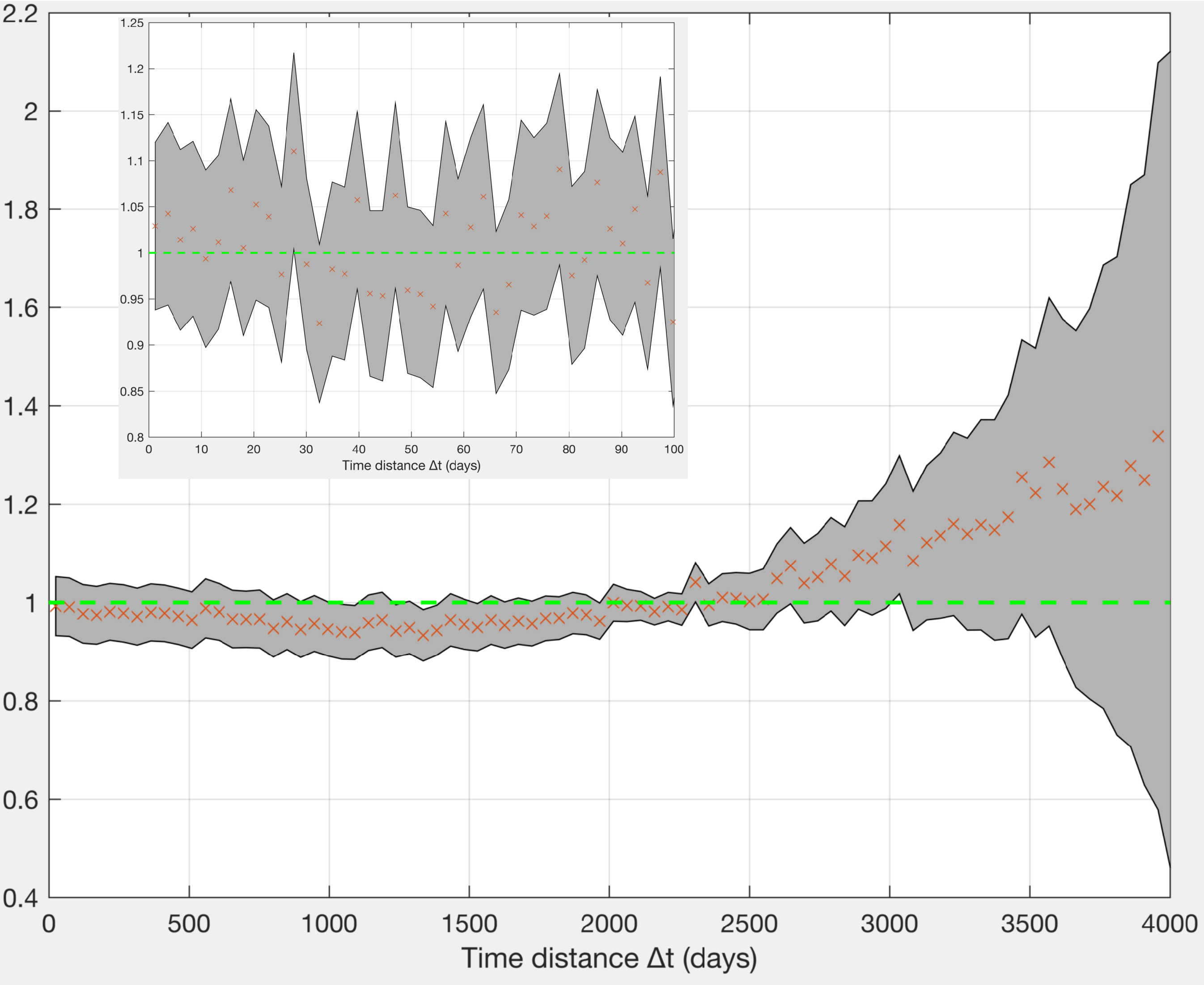

Supplementary Figure S3: Temporal pair distribution function. The plot shows the temporal pair distribution function (orange crosses) of the railway suicide cases, not taking the spatial information into account. The dashed green reference line represents a baseline Poisson process with a rate of 1130 cases over 4370 days $\approx$ 0.26 cases per day. The inset shows a close-up of the first 100 days. The gray simulation envelopes are calculated as the point-wise mean standard deviation of 1000 realizations of the baseline Poisson process. The temporal distribution function shows no signs of temporal clustering.
